# Gui‐Shao San Umbilical Moxibustion for Diarrhea‐Predominant Irritable Bowel Syndrome With Liver Depression and Spleen Deficiency Pattern: A Randomized Controlled Trial

**DOI:** 10.1155/grp/1832176

**Published:** 2026-05-18

**Authors:** Mengyan Zhou, Yulu He, Longshu Zhang, Junquan Xia, Zhentao An, Xixia Zhang

**Affiliations:** ^1^ School of Nursing, Nanjing University of Chinese Medicine, Nanjing, Jiangsu, China, njucm.edu.cn; ^2^ Affiliated Hospital of Integrated Traditional Chinese and Western Medicine, Nanjing University of Chinese Medicine, Nanjing, Jiangsu, China, njucm.edu.cn

## Abstract

**Objectives:**

This randomized controlled trial aimed to evaluate the effects of Gui‐Shao San umbilical moxibustion on symptoms of the disease, psychological well‐being, and quality of life in patients with IBS‐D.

**Interventions:**

The patients were randomly assigned to three groups: One received Gui‐Shao San umbilical moxibustion alongside conventional Western medicine treatment; one received placebo umbilical moxibustion alongside conventional Western medicine treatment; and one received only conventional Western medicine treatment for use as a positive control group.

**Outcomes:**

The primary outcome measures included TCM clinical symptom score, IBS‐SSS, and grading criteria for TCM syndrome scores. Secondary outcome measures included SAS, SDS, and IBS‐QOL. The effects of the intervention on these outcomes were measured at three time points: baseline (T0), Week 2 (T1), and Week 4 (T2). At Week 8, the recurrence of patients was measured based on whether their IBS‐SSS score increased by one grade

**Results:**

After 4 weeks of treatment, compared with the positive drug control group, both the Gui‐Shao San umbilical moxibustion group and the placebo umbilical moxibustion group effectively relieved patients′ symptoms, anxiety, and depression, while improving their quality of life. Significant differences were observed between groups in IBS‐SSS scores (120.61 ± 30.61 vs. 128.13 ± 41.85 vs. 161.82 ± 59.87, *p* < 0.001) and TCM syndrome scores (7.88 ± 1.709 vs. 8.59 ± 2.722 vs. 9.97 ± 2.099, *p* < 0.001).

**Conclusions:**

Gui‐Shao San umbilical moxibustion is an effective method for treating IBS‐D. Further rigorous, large‐scale, long‐term trials are needed to evaluate the therapeutic effects of Gui‐Shao San umbilical moxibustion, focusing on interdisciplinary collaboration.

**Trial Registration:**

The International Traditional Medicine Clinical Trial Registry (ITMCTR) Reg. No.: ITMCTR2025001072

## 1. Introduction

Irritable bowel syndrome (IBS) is an inveterate and burdensome gastrointestinal disease characterized by abdominal pain associated with alteration of bowel habits [1]. The prevalence varies considerably from country to country. The prevalence of IBS in the United States, the United Kingdom, and Canada is approximately 4.4%–4.8% [2]. The overall prevalence of IBS in China ranges from 1.4% to 11.5%, with it being more common in young and middle‐aged people (aged 18–59 years) [3]. Diarrhea‐predominant irritable bowel syndrome (IBS‐D), the most common subtype in the Rome IV criteria, accounts for 31.5% of all IBS patients [4]. Clinical studies have shown that liver depression and spleen deficiency are the major syndromes in IBS‐D patients, accounting for 44.7% of the cases [5]. People with IBS have a severe disease burden and unmet needs, and their work and activities of daily living can be severely affected [6]. Irritable bowel is mostly accompanied by anxiety and depression. This seriously affects people′s quality of life and mental health; furthermore, IBS places a huge financial burden on patients and the healthcare system [7].

The etiology and pathogenesis of IBS‐D remain to be fully elucidated. The prevailing hypothesis suggests that this condition is predominantly associated with compromised intestinal barrier function, dysbiosis of intestinal microbiota, visceral hypersensitivity, and psychological distress [8–11]. In traditional Chinese medicine (TCM), IBS‐D is categorized as a disorder characterized by abdominal discomfort and diarrhea, often comorbid with other diseases. Its pathogenesis is primarily attributed to dysfunction of the liver, spleen (stomach), and kidneys. The predominant pathomechanism involves liver depression and spleen deficiency: When impaired hepatic function and deficient splenic transport occur, water and dampness accumulate and overflow into the intestinal tract, leading to diarrhea. The principal therapeutic strategy for IBS‐D in TCM focuses on fortifying the spleen and regulating the liver [12].

Given the complex pathophysiology of IBS‐D, there is currently no definitive cure for this condition. Current treatment options for IBS‐D are limited to symptomatic management, necessitating individualized therapeutic approaches. Although three FDA‐approved medications have demonstrated efficacy in alleviating IBS‐D symptoms, they do not provide a complete cure [13]. Due to the chronic nature of the disorder, patients often pursue nonpharmacological interventions, such as dietary modifications, physical activity, and cognitive behavioral therapy. However, the available evidence supporting these treatments remains inconclusive, necessitating further investigation before their routine clinical application. TCM offers alternative therapeutic modalities for IBS‐D, including oral herbal formulations, acupuncture, acupressure, and adjunctive therapies [14–16]. Nevertheless, oral herbal medicine is associated with potential adverse effects, including gastrointestinal disturbances, dermatological reactions, and hepatotoxicity [17]. Acupuncture, though effective, is an invasive procedure requiring administration by certified practitioners. Acupressure, a noninvasive technique involving acupoint stimulation, can be self‐performed but demands prolonged training to ensure accurate point selection [16]. In contrast, moxibustion has shown promise in managing IBS‐D, offering a safe, accessible, and cost‐effective therapeutic option [18].

Umbilical moxibustion is a specialized form of moxibustion therapy in TCM. Based on TCM theory and syndrome differentiation, medicinal substances are formulated into specific preparations (e.g., ointments or powders) and applied to the umbilicus (Shenque acupoint). This is combined with moxibustion to achieve therapeutic effects. The Shenque acupoint, located at the center of the navel, is the primary target of umbilical moxibustion. According to TCM meridian theory, the Shenque acupoint is closely associated with the spleen, stomach, and large intestine. The periumbilical region has relatively thin subcutaneous fat and stratum corneum, along with abundant vascularization. The concave structure of the navel facilitates drug retention and enhances transdermal absorption, further supporting the therapeutic mechanism of umbilical moxibustion. Moxibustion is a therapeutic technique that involves the burning of moxa, a medicinal herb derived from *Artemisia argyi*, at specific acupuncture points or body surface areas to stimulate physiological regulation. Numerous studies have confirmed its clinical efficacy in treating various conditions, including rheumatoid arthritis, insomnia, and diabetic peripheral neuropathy [19–21].

Gui‐Shao San (Patent No. ZL201310479950.2) is a traditional Chinese medicinal formulation composed of a diverse array of botanical ingredients, including Radix Paeoniae Alba, cinnamon, *Asarum*, Radix Angelicae Dahuricae, Rhizoma Zingiberis, frankincense, costustoot, and borneol. In TCM, the root of Radix Paeoniae Alba is recognized for its ability to nourish yin, soften the liver, and alleviate pain. Pharmacological studies have demonstrated its anti‐inflammatory, analgesic, and antithrombotic effects. Cinnamon exhibits antidiarrheal and analgesic properties. *Asarum* has been reported to possess analgesic activity [22]. Similarly, Radix Angelicae Dahuricae displays a broad spectrum of pharmacological effects, including analgesic, antispasmodic, anti‐inflammatory, antipyretic, and anticancer actions [23]. Rhizoma Zingiberis, also known as concocted ginger, is a traditional Chinese medicinal preparation with demonstrated analgesic effects in pharmacological studies. In TCM, Rhizoma Zingiberis is indicated for warming the middle jiao and dispelling cold [24]. Frankincense exhibits blood‐activating properties, alongside anti‐inflammatory and analgesic effects [25]. Costustoot has been reported to alleviate pain, strengthen the spleen, and resolve food stagnation. Borneol has been demonstrated to improve the transdermal absorption of drugs [23]. Collectively, these components synergistically promote hepatic function, fortify the spleen, warm the middle jiao, relieve pain, and regulate intestinal motility to alleviate diarrhea.

The intervention employed the therapeutic modality of umbilical cord moxibustion combined with Gui‐Shao San, a multimodal approach integrating moxibustion, acupoint stimulation, and transdermal drug administration, which is hypothesized to exert a synergistic therapeutic effect. Owing to the unique characteristics of umbilical moxibustion, this study utilizes a single‐blind design to assess the clinical efficacy of this intervention. Moreover, existing literature lacks sufficient evidence to guide individualized treatment strategies for patients. This trial aims to address this knowledge gap by implementing a methodologically rigorous approach to evaluate the treatment efficacy in patients with IBS‐D characterized by liver depletion and spleen deficiency. The present randomized controlled study examined the effects of Gui‐Shao San on the primary and secondary outcomes in patients with IBS‐D with liver depression and spleen deficiency. The primary outcomes included the TCM symptom score and the Irritable Bowel Syndrome Symptom Severity Score (IBS‐SSS), whereas the secondary outcomes comprised the TCM gastrointestinal score, Self‐Rating Anxiety Scale (SAS), and Self‐Rating Depression Scale (SDS). The objective of this study was to compare three groups: the Gui‐Shao San group, the placebo group, and the positive control group, to evaluate the efficacy of umbilical cord moxibustion in the treatment of IBS‐D with liver depression and spleen deficiency. Additionally, this study is aimed at assessing whether a combined approach of Chinese and Western medicine could provide superior therapeutic effects and improve the quality of life in IBS‐D patients.

## 2. Materials and Methods

### 2.1. Study Design

This was a single‐center, single‐blind, placebo‐controlled, randomized controlled trial, which was approved by the Ethics Committee of Jiangsu Provincial Hospital of Integrative Medicine (Ethics No. 2025‐LWKYZ‐016) and registered on the website of the International Center for Clinical Trials (Reg. No. ITMCTR2025001072). The study was conducted in accordance with the Consolidated Standards of Reporting Trials (CONSORT) guidelines. The trial was carried out in the outpatient clinic or ward of the Department of Gastroenterology, Jiangsu Provincial Hospital of Integrative Medicine. Before enrollment, all participants received a comprehensive explanation of the study′s objectives, methods, potential risks, and benefits.

### 2.2. Sample Size

This study is a randomized controlled trial, with subjects randomly assigned to three groups. The primary outcome measure was the total IBS‐SSS score at 4 weeks postintervention. Based on a review of relevant literature and preliminary pilot study results [26], the expected mean total IBS‐SSS scores at 4 weeks postintervention are 154.41, 122.06, and 162.94, respectively, with standard deviations of 31.19, 26.96, and 42.95. A two‐tailed test was required, with *α* set at 0.05 and a power level of 90%. Using PASS 15 software, the sample size per group was calculated as *N* = 27. Accounting for a 20% attrition rate due to loss to follow‐up or refusal to participate, each group ultimately required a minimum of 34 participants, totaling 102 subjects for the study.

### 2.3. Participants and Sampling

The following are the criteria that have been included: Participants must voluntarily participate in this clinical study and sign an informed consent form. They must also be patients with IBS‐D who meet the Rome IV criteria and the criteria for the liver qi stagnation and spleen deficiency type of IBS‐D, as defined in the “Expert Consensus on the Diagnosis and Treatment of IBS in TCM,” which was approved by the Gastroenterology Branch of the Chinese Association of Traditional Chinese Medicine in 2024. Participants must also be conscious and aged between 18 and 59 years.

The following were considered as exclusion criteria: (1) Individuals with concurrent organic gastrointestinal tract lesions, such as moderate‐to‐severe peptic ulcers, erosions, neoplasms, intestinal epithelial metaplasia, or heterotopia; (2) individuals with concurrent severe cardiovascular, cerebrovascular, hepatic, renal, or other primary diseases, including coagulation dysfunction and cancer patients; (3) individuals with psychiatric illnesses or severe neuroses; (4) women who are pregnant, lactating, or planning to become pregnant; (5) individuals who had taken antibiotics, probiotic preparations, or gastrointestinal stimulant drugs within 1 month prior to trial enrollment; (6) individuals with poor adherence to medical advice and intolerance to moxibustion; (7) individuals who were allergic to the ingredients of the medicinal powder; (8) individuals who were participating in other clinical studies.

### 2.4. Randomization and Blinding

The study employed block randomization to form groups, with six participants per block. This setup created 90 possible sequences for the GU (A), PU (B), and PC (C) groups within each block. These sequences were assigned numbers 1–90, requiring 17 complete blocks based on the total sample size and block length. Using Excel, 17 random integers from 1 to 90 were generated and placed into 17 sealed opaque envelopes. A researcher not involved in the intervention process sequentially opened the envelopes and assigned the six participants in each block to the corresponding group (A, B, or C) according to the random number sequence.

Since the control group did not receive umbilical moxibustion, this study could not be blinded. To mitigate any potential bias, the powder was prepared by professionals and labeled with serial numbers. The operators performing umbilical moxibustion, the evaluators of the results, and the statisticians were unaware of the allocation of each patient to a group.

### 2.5. Intervention

Patients in the PC group were routinely prescribed oral administration of trimebutine maleate capsules (specification: 0.1 g; National Drug Approval Number H20040713, Shanxi Zhendong Ante Biological Pharmaceutical Co. Ltd.). The capsules should be taken according to the recommendations of medical professionals, three times a day, one capsule each time. Concurrently, routine nursing interventions were implemented: (1) Health education: the improvement of patients′ health education to enhance their understanding of the condition. (2) Lifestyle and social behavior modifications: curtail tobacco and alcohol consumption, ensuring adequate rest and sleep, avoiding prolonged excessive fatigue, and paying special attention to lifestyle adjustments in winter and spring to prevent colds. Regular exercise, including TCM health exercises like Tai Chi, is also important. (3) Dietary care: avoiding consuming high‐fat, spicy, numbing, and heavily seasoned foods. (4) Psychological care: maintaining a positive mood, developing a good outlook on life, and avoiding emotional distress.

The subjects in the GU group received moxibustion on the navel using Gui‐Shao San, a drug ground into powder and sieved through a 100‐mesh screen. This treatment was administered in addition to the PC group′s treatment.

The PU group received moxibustion on the navel using a placebo (each dose contained 4% crude drug, with food coloring and starch added in appropriate amounts, prepared according to the method for Gui‐Shao San, resulting in a simulated agent identical in appearance, size, and dose to Gui‐Shao San) in addition to the PC group′s treatment.

The intervention was performed by a dedicated operator who had undergone training in the operating procedures and acupoint localization and passed the assessment. Prior to undergoing the procedure, the operator was responsible for explaining the purpose of the study and the precautions to the patient.

Researchers guided participants to receive two weekly moxibustion treatments at the navel. The specific operation was as follows: (1) Preparation of the bowl: The operator mixed flour with water to form a dough ball (8 cm in diameter, 2 cm thick, with a central hole 2.5 cm in diameter). (2) Procedure: After the participant voids, they lay flat on their back. The operator laid a perforated cloth on the abdomen, placed the navel bowl over the navel, and filled it with 8–10 g of herbal powder. Moxibustion was performed using moxa cones (3.3 cm in diameter, 2.6 cm in height) placed over the powder. Three consecutive moxa cones were ignited and positioned above the medicinal hole (when adding new cones, the burning ones were used to blow away ash before placement). Each session lasted approximately 20 min, totaling 60 min. After treatment, the herbal powder was secured with a medical‐grade polyurethane navel patch for 4 h. The entire course was administered twice weekly for 4 weeks [27].

In order to enhance participant compliance, trainers made contact with participants online on a weekly basis and shared knowledge related to the disease. Follow‐up assessments were conducted in Weeks 2 and 4.

Throughout the study, participants may take emergency medication as directed if they have uncontrollable diarrhea or other discomfort. If a patient introduced any new medications for IBS‐D treatment for more than 3 days, which compromised the assessment of the study drug′s efficacy, this was considered a protocol violation.

### 2.6. Outcome Measurements

It is essential to collect patients′ baseline characteristics during their initial visit, including gender, age, body mass index (BMI), educational level, perceived stress levels, regular exercise habits (defined as exercising more than four times per week), and disease duration.

### 2.7. TCM Clinical Symptom Score [28]

In accordance with the Guidelines for Clinical Research on New Traditional Chinese Medicines (Interim), five symptoms including abdominal pain, abdominal distension, frequency of bowel movements, stool consistency, and sensation of incomplete bowel movement were scored before and after treatment. The manifestation of symptoms was categorized according to severity as follows: *none*, *mild*, *moderate*, or *severe*, and scores of 0, 1, 2, and 3 were assigned to them, respectively.

### 2.8. The IBS‐SSS [29]

The scoring table comprises five sections: severity of abdominal pain, time of occurrence of abdominal pain within 10 days, discomfort caused by abdominal pain, satisfaction with bowel movements, and impact on quality of life. Each section of the scoring table is assigned a score from 0 to 100 points, and the total score ranges from 0 to 500 points. A higher score on this scale indicates a more severe condition.

### 2.9. Grading Criteria for TCM Syndrome Scores [28]

It is imperative to observe the primary symptoms, which include abdominal pain followed by diarrhea, with pain being alleviated after diarrhea; irritability and anger; and frequent sighing. The secondary symptoms to be observed are distension of the flanks, loss of appetite, fatigue, and weakness. In addition to these, other symptoms and signs must be noted. The primary symptoms are evaluated on a four‐tier scale, ranging from *absent* (Score 0) to *severe* (score 6), with intermediate categories of *mild* (Score 2) and *moderate* (Score 4). The secondary symptoms are scored according to four levels: *absent*, *mild*, *moderate*, and *severe*, with scores of 0, 1, 2, and 3, respectively. The total syndrome score is derived by calculating the sum of the scores for each individual symptom of the aforementioned TCM syndromes.

### 2.10. SAS [30] and SDS [31]

Both scales consist of 20 items, and each item is scored on a 1‐ to 4‐point scale. The cumulative scores are derived from the raw scores of SAS and SDS. Subsequently, the raw scores are multiplied by 1.25 and rounded to the nearest whole number, thereby yielding the standardized scores of the SAS and the SDS. For the SAS, scores below 50 are considered normal. Scores ranging from 50 to 59 indicate mild anxiety, those from 60 to 69 indicate moderate anxiety, and scores of 70 or above indicate severe anxiety. Similarly, for the SDS, scores below 50 are classified as normal. Scores ranging from 50 to 59 indicate mild depression, those from 60 to 69 indicate moderate depression, and scores of 70 or above indicate severe depression.

### 2.11. Irritable Bowel Syndrome Quality of Life Scale (IBS‐QOL) [32]

This scale assesses patients′ quality of life, including eight dimensions: anxiety and restlessness, behavioral disorders, body awareness, health concerns, dietary restrictions, social reactions, relationships with the opposite sex, and interpersonal relationships. Each dimension is scored from 0 to 100, with a total score ranging from 0 to 800. A higher score indicates that IBS has less impact on the quality of life.

### 2.12. Long‐Term Efficacy

Using IBS‐SSS as the primary endpoint, the recurrence rate at Week 8 posttreatment was observed in the three groups of patients, with primary symptoms and scores recorded. An elevation of one grade in symptom scores from the initial baseline measurement was designated as recurrence, and the recurrence rate was subsequently calculated. The recurrence rate, expressed as a percentage, was calculated by dividing the number of recurrences by the total number of effective cases and multiplying the result by 100.

### 2.13. Safety Assessment

Patients should be informed of potential adverse reactions such as skin redness, mild burns, localized itching, and peeling before treatment. Throughout the intervention process, all adverse reactions should be systematically recorded and assessed. If any adverse reactions occur, the occurrence time, severity, duration, and management approach should be documented promptly. In cases of severe or intolerable symptoms, treatment should be immediately discontinued and appropriate measures taken. This monitoring process should be maintained throughout the entire treatment course to ensure the safety evaluation of moxibustion therapy.

## 3. Data Analysis

The analysis was conducted using IBM SPSS 27.0 software. In the field of descriptive statistics, qualitative variables were represented by percentages and frequencies, whereas continuous variables were represented by means and standard deviations. The Shapiro–Wilk test was employed to evaluate the normality of numerical variables, whereas Levene′s test was used to assess the homogeneity of variances. Chi‐square tests were used to determine the significance of changes in binary and multinomial variables, respectively. For between‐group comparisons, parametric data were analyzed using the analysis of variance (ANOVA) test, whereas nonparametric data were analyzed using the Kruskal–Wallis (K–W) test.

After conducting normality tests on all data, for normally distributed data, repeated measures ANOVA was employed to evaluate the main effects of time and grouping as well as their interaction effects. The sphericity hypothesis was tested using Moch′s test, with the “assumed sphericity” result applied when *p* > 0.05; conversely, when *p* < 0.05, the Greenhouse–Geisser (G–G) correction was used to adjust the within‐subject effects. When main effects or interaction effects were significant, Bonferroni correction was applied for necessary post hoc pairwise comparisons. For nonnormally distributed data, the Friedman test was used for between‐group comparisons, generalized estimating equations (GEEs) were used for within‐group comparisons, and Bonferroni‐corrected post hoc pairwise comparisons were conducted to examine specific differences.

## 4. Results

### 4.1. Characteristics of Participants

**Figure 1:**
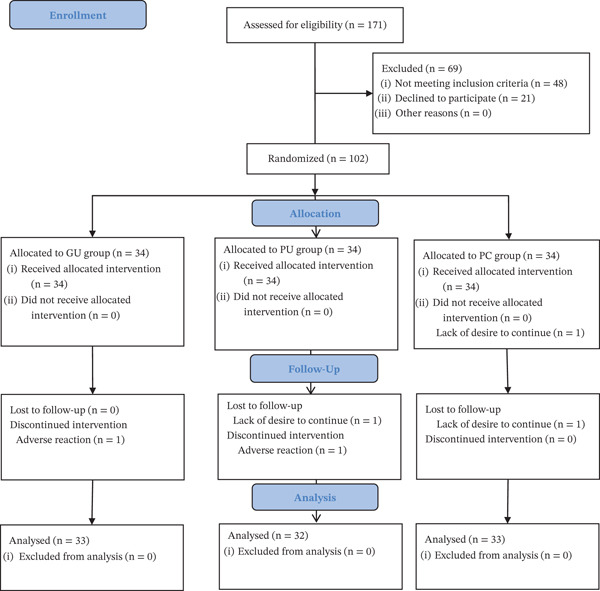
Flow diagram of the study.

As demonstrated in Figure 1, the study was conducted from March 2025 to July 2025, during which a total of 171 participants were screened for eligibility. Of these, 48 were excluded on the basis that they did not meet the inclusion criteria, 21 refused to participate, and the remaining 102 patients were randomly assigned to three groups. A total of 98 patients (33 in the GU group, 32 in the PU group, and 33 in the PC group) completed the trial and were included in the analysis.

As demonstrated in Table 1, the mean age of subjects in the GU group was 43.09 ± 10.572, the mean age of subjects in the PU group was 41.13 ± 11.826, and the mean age of subjects in the PC group was 42.36 ± 11.437. The majority of participants in all three groups were middle‐aged and young adults. The majority of participants were female (60.6% in the GU group, 53.1% in the PU group, and 54.5% in the PC group). With regard to educational attainment, the majority of participants had obtained an associate degree or higher qualification. With regard to BMI, the majority of participants in all three groups were classified as overweight or obese (75.8% in the GU group, 65.7% in the PU group, and 72.7% in the PC group). In terms of disease duration, the average disease duration for participants in the GU group was 6.39 ± 3.807, that for the PU group was 6.56 ± 2.918, and that for the PC group was 6.15 ± 4.101. With regard to physical activity, 63.6% of patients in the GU group, 50% in the PU group, and 54.5% in the PC group reported enjoyment of exercise. With regard to stress, participants in all three groups reported feeling significant stress from life or work (66.7% in the GU group, 53.1% in the PU group, and 60.6% in the PC group). The baseline demographic and clinical characteristics of the three groups were fully matched, and all comparisons yielded *p* > 0.05.

**Table 1:** Sociodemographic and clinical characteristics of the subjects.

Characteristics	GU group (*n* = 33)		PU group (*n* = 32)		PC group (*n* = 33)		Test statistic	*p*
	*n*	%	*n*	%	*n*	%		
Age (*X* ± SD)	43.09 ± 10.572		41.13 ± 11.826		42.36 ± 11.437		*F* = 0.251	0.778
18–29	4	12.1	5	15.6	6	18.2		
30–39	8	24.3	10	31.3	7	21.2		
40–49	10	30.3	8	25.0	11	33.3		
50–59	11	33.3	9	28.1	9	27.3		
Gender							*χ* ^2^ = 0.419	0.811
Male	13	39.4	15	46.9	15	45.5		
Female	20	60.6	17	53.1	18	54.5		
Education level							*χ* ^2^ = 1.858	0.932
Primary and lower education	2	6.1	1	3.1	1	3.0		
Junior high school education	8	24.2	6	18.8	5	15.2		
High school/junior college education	10	30.3	11	34.3	10	30.3		
College/undergraduate degree and above	13	39.4	14	43.8	17	51.5		
Body mass index (*X* ± SD)	24.89 ± 2.723		25.08 ± 3.153		24.83 ± 2.915		*H* = 0.162	0.922
Healthy weight	8	24.2	11	34.4	9	27.3		
Overweight	20	60.6	14	43.8	17	51.5		
Obese	5	15.2	7	21.9	7	21.2		
Course of disease (*X* ± SD)	6.39 ± 3.807		6.56 ± 2.918		6.15 ± 4.101		*H* = 0.941	0.625
0–5	17	51.5	13	40.6	17	51.5		
6–10	10	30.3	16	50.0	12	36.4		
> 10	6	18.2	3	9.4	4	12.1		
Exercise or not							*χ* ^2^ = 1.277	0.528
Yes	21	63.6	16	50	18	54.5		
No	12	36.4	16	50	15	45.5		
Press							*χ* ^2^ = 1.247	0.536
Mild	11	33.3	15	46.9	13	39.4		
Heavy	22	66.7	17	53.1	20	60.6		

Abbreviations: *F*, analysis of variance (ANOVA); GU group, Gui‐Shao San umbilical moxibustion group; *H*, Kruskal–Wallis (K–W) test; PC group, positive drug control group; PU group, placebo umbilical moxibustion group; *χ* ^2^, chi‐square test.

### 4.2. Comparative Effects of Three Groups on Outcome Measures

**Figure 2:**
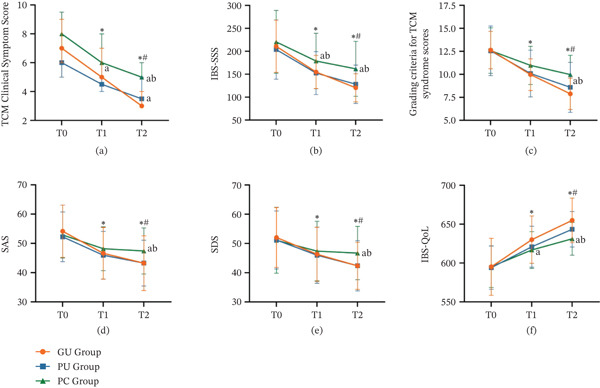
Trends in outcome measures during the treatment period and the follow‐up period. (a) Change in the curve of TCM Clinical Symptom Score within 4 weeks. (b) Change in the curve of IBS‐SSS within 4 weeks. (c) Change in the curve of Grading criteria for TCM syndrome scores within 4 weeks. (d) Change in the curve of SAS within 4 weeks. (e) Change in the curve of SDS within 4 weeks. (f) Change in the curve of IBS‐QOL within 4 weeks.

Table 2 shows the comparison of study results between groups at different time points; Table 3 shows the analysis of study results at different time points using repeated measures ANOVA; Table 4 shows the long‐term efficacy of the subjects; and Figure 2 shows the trend of changes in outcome measures during treatment period and follow‐up period.

**Table 2:** Between‐group comparisons of findings at different time points.

Outcome Measure‐mengts	Measure‐ment time	GU group (n=33)	PU group (n=32)	PC group (n=33)	Test statistic	*p*
TCM Clinical Symptom Score	T0	7(6,9)	6(5,9)	8(5,9.5)	1.125	0.57
	T1	5(5,7) ^∗^	4.5(4,7) ^∗^	6(4.5,8)a ^∗^	4.163	0.125
	T2	3(3,4) ^∗^^#^	3.5(3,5)a ^∗^^#^	5(4,6)ab ^∗^^#^	27.679	0.001
IBS‐SSS	T0	210.91±57.03	203.75±64.55	220.61±68.46	0.577	0.563
	T1	154.85±36.07 ^∗^	152.5±46.77 ^∗^	178.79±60.40ab ^∗^	2.914	0.059
	T2	120.61±30.61 ^∗^^#^	128.13±41.85 ^∗^^#^	161.82±59.87ab ^∗^^#^	7.572	0.001
Grading criteria for TCM syndrome scores	T0	12.64±2.028	12.56±2.699	12.58±2.475	0.009	0.991
	T1	9.97±1.723 ^∗^	10.09±2.545 ^∗^	10.97±2.084 ^∗^	2.089	0.129
	T2	7.88±1.709 ^∗^^#^	8.59±2.722 ^∗^^#^	9.97±2.099ab ^∗^^#^	7.622	0.001
SAS	T0	54.15±8.948	52.25±8.512	52.88±7.865	0.428	0.653
	T1	46.61±8.853 ^∗^	45.94±8.128 ^∗^	48.18±7.481 ^∗^	0.649	0.525
	T2	43.24±9.354 ^∗^^#^	43.28±7.817 ^∗^^#^	47.42±7.874ab ^∗^^#^	2.698	0.073
SDS	T0	52.06±10.238	51.19±9.911	51.09±11.282	0.085	0.918
	T1	46.33±9.299 ^∗^	45.97±9.573 ^∗^	47.39±10.189 ^∗^	0.191	0.827
	T2	42.36±8.093 ^∗^^#^	42.38±8.609 ^∗^^#^	46.73±9.105ab ^∗^^#^	2.803	0.066
IBS‐QoL	T0	595.15±36.668	594.09±27.85	595.3±26.749	0.015	0.985
	T1	630.03±30.474 ^∗^	620.97±26.31 ^∗^	616.79±23.631a ^∗^	2.079	0.131
	T2	654.88±28.624 ^∗^^#^	643.47±22.843 ^∗^^#^	631.15±20.999ab ^∗^^#^	7.812	0.001

*Note:* Pairwise comparisons were performed of each treatment group: Compared with the GU Group; a *p* < 0.05.Compared with the PU Group , b *p* < 0.05 ; Compared with T0,  ^∗^ *p* < 0.05; Compared with the T1, ^#^ *p* < 0.05.

Abbreviations: GU group: Gui‐shao San umbilical moxibustion group; PU group: Placebo umbilical moxibustion group; PC group: Positive drug control group; TCM: Traditional Chinese Medicine; IBS‐SSS: The Irritable Bowel Syndrome Symptom Severity Score; SAS: Self‐Rating Anxiety Scale; SDS: Self‐Rating Depression Scale; IBS‐QOL: Irritable Bowel Syndrome Quality of Life Scale. T0: Baseline, T1: Week 2, T2: Week 4.

**Table 3:** Within‐group comparisons of findings at different time points.

Outcome measurement	Time effect		Intergroup effect		Interactive effect	
	*F*	*p*	*F*	*p*	*F*	*p*
TCM clinical symptom score (Wald *χ* ^2^)	506.605	< 0.001	4.891	0.087	50.31	< 0.001
IBS‐SSS	321.767	< 0.001	2.753	0.069	4.677	0.008
Grading criteria for TCM syndrome scores	505.359	< 0.001	1.953	0.147	14.095	< 0.001
SAS	307.321	< 0.001	0.698	0.5	10.376	< 0.001
SDS	425.709	< 0.001	0.359	0.699	20.064	< 0.001
IBS‐QOL	574.657	< 0.001	1.819	0.168	12.016	< 0.001

Abbreviations: *F*, analysis of variance (ANOVA); IBS‐QOL, Irritable Bowel Syndrome Quality of Life Scale; IBS‐SSS, irritable Bowel Syndrome Symptom Severity Score; SAS, Self‐Rating Anxiety Scale; SDS, Self‐Rating Depression Scale; TCM, traditional Chinese medicine; Wald *χ* ^2^, generalized estimating equations.

**Table 4:** Teletherapy efficacy in subjects.

Outcomes	GU group (*n* = 33)		PU group (*n* = 32)		PC group (*n* = 33)		Test statistic	*p*
	*n*	%	*n*	%	*n*	%		
Recurrence or not							196.000	< 0.001
Yes	4	12.1	7	21.9	15	45.5		
No	29	87.9	25	78.1	18	54.5		

Abbreviations: GU group, Gui‐Shao San umbilical moxibustion group; PC group, positive drug control group; PU group, placebo umbilical moxibustion group.

### 4.3. Primary Outcome Measures

At baseline, the TCM clinical symptom score was 7 (6, 9) in the GU group, 6 (5, 9) in the PU group, and 8 (5, 9.5) in the PC group; no statistically significant differences were observed between the three groups. In Week 2, the TCM clinical symptom scores were 5 (5, 7) in the GU group, 4.5 (4, 7) in the PU group, and 6 (4.5, 8) in the PC group; no statistically significant differences were observed between the three groups. By Week 4, the TCM clinical symptom scores in all three groups had decreased significantly compared with baseline and Week 2. The TCM symptom scores were 3 (3, 4) in the GU group, 3.5 (3, 5) in the PU group, and 5 (4, 6) in the PC group. The TCM symptom scores in both the GU and PU groups were significantly lower than those in the PC group, with the GU group′s score being lower than that of the PU group; there was a statistically significant difference between the three groups (*p* < 0.05).

GEEs demonstrated that the TCM clinical symptom scores of the three groups varied significantly over time (Wald *χ* ^2^: 506.605, *p* < 0.001). Furthermore, at varying time points, the TCM clinical symptom scores of patients within different groups exhibited significant differences (Wald *χ* ^2^: 50.31, *p* < 0.001).

At baseline and Week 2, there was no statistically significant difference in the severity of IBS symptoms between the GU group (T1: 154.85 ± 36.07), the PU group (T0: 203.75 ± 64.55, T1: 152.5 ± 46.77), and the PC group (T0: 220.61 ± 68.46, T1: 178.79 ± 60.40), with most participants in all groups exhibiting moderate severity. By Week 4, the severity of symptoms in the GU group (120.61 ± 30.61), PU group (128.13 ± 41.85), and PC group (161.82 ± 59.87) showed statistically significant differences in symptom severity (*p* < 0.05). Specifically, at Weeks 2 and 4, the GU group and PU group had significantly lower IBS symptom severity than the PC group. According to descriptive statistics, the GU group exhibited a more pronounced downward trend compared to the PC group (T0–T2).

A repeated measures ANOVA was conducted, and it revealed significant variations in symptom severity among the three groups over time (*F* = 321.767, *p* < 0.001). Additionally, no substantial differences in symptom severity were observed between the three groups (*F* = 2.753, *p* = 0.069). Furthermore, significant variations in symptom severity were identified among the different groups at distinct time points (*F* = 4.677, *p* = 0.008).

At baseline and Week 2, the GU group (T0: 12.64 ± 2.028, T1: 9.97 ± 1.723), the PU group (T0: 12.56 ± 2.699, T1: 10.09 ± 2.545), and the PC group (T0: 12.58 ± 2.475, T1: 10.97 ± 2.084) demonstrated no statistically significant variations in TCM syndrome scores. By Week 4, a significant decrease in TCM syndrome scores was observed in the GU group (7.88 ± 1.709), the PU group (8.59 ± 2.722), and the PC group (9.97 ± 2.0999) compared to baseline and Week 2. A statistically significant difference was found among the three groups (*p* < 0.05). At Week 4, the TCM syndrome scores of the GU and PU groups were found to be lower than those of the PC group. According to descriptive statistics, the GU group exhibited a more pronounced downward trend compared to the PC group (T0–T2).

A repeated measures ANOVA was conducted, revealing that the TCM syndrome scores of the three groups exhibited a significant time‐related variation (*F* = 505.359, *p* < 0.001). However, no substantial differences were observed in TCM syndrome scores among the three groups (*F* = 1.953, *p* = 0.147). Nevertheless, a clear distinction in TCM syndrome scores was identified among different groups at varying time points (*F* = 14.095, *p* < 0.001).

### 4.4. Secondary Outcome Measures

At baseline, patients in the GU group (T0: 54.15 ± 8.948), the PU group (T0: 52.25 ± 8.512), and the PC group (T0: 52.88 ± 7.865) all experienced mild anxiety. At the T1 and T2 time points, the GU group (T1: 46.61 ± 8.853, T2: 43.24 ± 9.354), the PU group (T1: 45.94 ± 8.128, T2: 43.28 ± 7.817), and the PC group (T1: 48.18 ± 7.481, T2: 47.42 ± 7.874) all showed improvements in anxiety levels. There were no statistically significant differences at any of the three time points. Anxiety scores in all three groups decreased significantly over time (*F* = 307.321, *p* < 0.001). At Week 4, the SAS scores of the PC and PU groups were significantly lower than those of the GU group. At different time points, there were significant differences in anxiety scores between different groups (*F* = 10.376, *p* < 0.001).

At baseline, patients in the GU group (T0: 52.06 ± 10.238), the PU group (T0: 51.19 ± 9.911), and the PC group (T0: 51.09 ± 11.282) were all in a mildly depressed state. At the T1 and T2 time points, patients in the GU group (T1: 46.33 ± 9.299, T2: 42.36 ± 8.093), the PU group (T1: 45.97 ± 9.573, T2: 42.38 ± 8.609), and the PC group (T1: 47.39 ± 10.189, T2: 46.73 ± 9.105) showed improvements in depression. There were no statistically significant differences among the three time points, but depression scores in all three groups decreased significantly over time (*F* = 425.709, *p* < 0.001). However, there were significant differences in depression scores between different groups at different time points (*F* = 20.064, *p* < 0.001).

At baseline and Week 2, the GU group (T0: 595.15 ± 36.668, T1: 630.03 ± 30.474), the PU group (T0: 594.09 ± 27.85, T1: 620.97 ± 26.31), and the PC group (T0: 595.3 ± 26.749, T1: 616.79 ± 23.631) showed no statistically significant differences in quality of life scores. By Week 4, all three groups exhibited a significant increase in quality of life compared to baseline. The GU group (654.88 ± 28.624) and the PU group (643.47 ± 22.843) have significantly higher scores than those of the PC group (631.15 ± 20.999). The quality of life scores of all three groups significantly improved over time (*F* = 574.657, *p* < 0.001). At different time points, there were significant differences in quality of life scores between different groups (*F* = 12.016, *p* < 0.001).

Four weeks after treatment ended, the recurrence rate was 12.1% (4/33) in the GU group, 21.9% (7/32) in the PU group, and 45.5% (15/33) in the PC group. Statistically significant differences were detected between the groups: The recurrence rate in the GU group was lower than that in the control group (PU), and the recurrence rate in the PU group was lower than that in the PC group.

Additionally, two participants (one from the GU group and one from the PU group) reported skin allergic reactions. The adverse reaction rate was only 3.07% (2/65), and there were no serious adverse reactions. These cases were referred to a dermatologist for appropriate management.

## 5. Discussion

The findings of this study indicate that symptoms decreased gradually over time in all three treatment groups. However, the GU and PU groups demonstrate superior outcomes in alleviating disease symptoms and improving quality of life. Moreover, 4 weeks after treatment, recurrence rates were significantly lower in the GU and PU groups compared to the PC group. Collectively, these results suggest that umbilical moxibustion is an effective therapeutic approach for IBS characterized by liver depression and spleen deficiency.

The therapeutic effects of umbilical moxibustion may be attributable to the thermal stimulation from burning moxa wool, which activates TRP family ion channels and heat shock proteins (HSPs) on skin cells, thereby regulating functions of the nervous, immune, endocrine, and circulatory systems [33]. Previous studies have demonstrated that moxibustion therapy provides long‐term symptom relief for IBS‐D patients, including improved stool consistency, reduced bowel movement frequency, and alleviated urgency and that it is more efficacious than certain alternative therapies [34, 35]. In addition, the umbilical region (CV8, Shenque point) is easily localized. Provided standardized procedures and safety monitoring are in place, this modality could be explored as a potential home‐based treatment option for patients in the future.

Moreover, the study revealed no significant differences between the GU and PU groups in improving patients′ anxiety, depression, or quality of life. In contrast, the placebo exhibited measurable efficacy in alleviating IBS symptoms, a phenomenon that may be attributed to its impact on patients′ psychological state. Extant research suggests that stress has been shown to trigger IBS‐D symptoms and contribute to anxiety and depression [11]. Patients with strong confidence and positive expectations regarding treatment may experience reduced stress levels, thereby contributing to symptom alleviation. This phenomenon may also be associated with patients′ widespread acceptance of conventional medical therapies.

In previous clinical studies, the research team observed that the Gui‐Shao powder umbilical patch exhibited efficacy in the treatment of IBS‐D; however, its preparation process was found to be relatively complex. The present study was conducted to treat patients with IBS‐D of liver depression and spleen deficiency type using the Gui‐Shao powder umbilical patch, with the original ointment formulation replaced by a powder formulation. As demonstrated in the preceding section, the powder formulation offers several advantages, including maintained efficacy, dose adjustability based on symptom severity, and an increased contact area. Nevertheless, its transdermal absorption remains limited owing to the lack of penetration enhancers and nanocarriers.

Nevertheless, it is evident that both the GU and PU groups exhibited markedly superior efficacy to the PC group in ameliorating patients′ symptoms, psychological state, and quality of life. This finding aligns with the integrated Chinese–Western medicine approach in the treatment of liver qi stagnation and spleen deficiency–type IBS. For instance, Zhang et al. reported that integrated Chinese–Western medicine therapy (Anchang Decoction) significantly enhances intestinal mucosal barrier function, reduces inflammatory markers, and regulates gastrointestinal motility. The mechanism of action of these interventions is thought to result from a combination of effects, which collectively alleviate symptoms and target the underlying pathology [36].

This study also has certain limitations. Firstly, it was a single‐blind trial with a short observation period, which may affect the long‐term reliability of the results. Secondly, patients′ differing perceptions of the disease and attitudes towards life can lead to significant fluctuations in psychological factors, which can interfere with the accuracy of efficacy evaluations. Furthermore, studies related to drug formulations and transdermal absorption capacity need to be given more attention, as interdisciplinary collaboration could benefit patients. Finally, this study only included Chinese patients, who may be subject to cultural influences and the placebo effect. Future studies should consider conducting multicenter, large‐scale randomized double‐blind trials and including participants from different ethnic groups.

## 6. Conclusion

The results of this study suggest that umbilical moxibustion using Gui‐Shao San is effective in alleviating clinical symptoms, alleviating anxiety and depression, and improving quality of life in patients with IBS‐D. It has the advantages of low recurrence rate and low adverse reaction rate. It can therefore be considered a safe and effective intervention for managing IBS‐D symptoms. However, no significant difference was observed between the Gui‐Shao San and placebo groups. This suggests that future studies should conduct large‐sample, long‐term randomized controlled trials, combining interdisciplinary cooperation to explore drug formulation optimization and transdermal absorption mechanisms in depth. This would improve the accuracy and repeatability of treatment.

## Author Contributions

**Mengyan Zhou:** writing – original draft, writing – review and editing, conceptualization, data curation, investigation, methodology, validation, visualization. **Yulu He:** writing – original draft, writing – review and editing, conceptualization, data curation, investigation, methodology, validation. **Longshu Zhang:** conceptualization, data curation, investigation, validation, writing – original draft. **Junquan Xia:** conceptualization, data curation, investigation, methodology, project administration, resources. **Zhentao An:** formal analysis. **Xixia Zhang:** conceptualization, data curation, supervision, resources, funding acquisition, methodology, project administration.

## Funding

This study was supported by the Postgraduate Research & Practice Innovation Program of Jiangsu Province (SJCX25_0906) and Provincial Traditional Chinese Medicine Science and Technology Development Program Projects (MS2023034).

## Ethics Statement

This study was approved by the Ethics Committee of Jiangsu Provincial Hospital of Integrative Medicine (Ethics No. 2025‐LWKYZ‐016) and registered on the website of the International Center for Clinical Trials (Reg. No. ITMCTR2025001072) (2025‐03‐19).

## Conflicts of Interest

The authors declare no conflicts of interest.

## Data Availability

The data that support the findings of this study are available on request from the corresponding author. The data are not publicly available due to privacy or ethical restrictions.
